# A patient with glycogen storage disease type Ia combined with chronic hepatitis B infection: a case report

**DOI:** 10.1186/s12881-019-0816-9

**Published:** 2019-05-20

**Authors:** Wenying Wang, Rentao Yu, Wenting Tan, Yunjie Dan, Guohong Deng, Jie Xia

**Affiliations:** 1 Department of Infectious Diseases, Southwest Hospital, Army Medical University, Chongqing, 400038 China; 2 Chongqing Key Laboratory of Infectious Diseases, Southwest Hospital, Army Medical University, Chongqing, 400038 China; 3 Department of Respiratory, the General Hospital of Western Theater Command, Chengdu, 460000 China

**Keywords:** GSD Ia, G6PC gene, Growth retardation, Chronic hepatitis B

## Abstract

**Background:**

Glycogen storage disease type I (GSD I), also known as von Gierk disease, is a metabolic disorder leading to the excessive accumulation of glycogen and fat in organs, characterized by hepatomegaly, hypoglycemia, lactic acidemia, hyperlipidemia, hyperuricemia, puberty delay and growth retardation, which can be indicated by height, weight, blood glucose and blood lipids.

**Case presentation:**

Here we present a 16-year-old male patient with GSD Ia complicated with hepatic adenoma and combined with hepatitis B. As a chronic hepatitis B patient, the patient was admitted to hospital in order to further clarify the nature of hepatic space occupancy because of suspicion of hepatocellular carcinoma. However, the imaging studies did not support hepatocellular carcinoma certainly. And by tracing his clinical history, we suggested that he might suffer from GSD I. Finally the diagnosis was confirmed by MRI (Gd-EOB-DTPA), liver biopsy and whole exome sequencing (WES).

The WES discovered a homozygous point mutation at the exon 5 of G6PC gene at 17th chromosome, c.G648 T (p.L216 L, NM_000151, rs80356484). This pathogenic mutation causes CTG changing to CTT at protein 216. Though both codons encode leucine, this silent mutation creates a new splicing site 91 bp downstream of the authentic splice site. According to previous research, this mutation is a disease causal variant for GSD Ia, and has a high frequency among GSD patients in China and Japan.

This patient was finally diagnosed as GSD Ia complicated with hepatic adenoma and combined with chronic hepatitis B, and received corn starch therapy immediately after GSD was suspected.

After receiving corn starch therapy, the height and weight of the patient were increased, and the secondary sexual characteristics were developed, including beard, pubic hair and seminal emission. Unexpectedly, the liver adenomas were still increasing, and we did not find any cause to explain this phenomenon.

**Conclusion:**

This patient was diagnosed as GSD Ia combined with chronic hepatitis B, who responded to corn starch intervention. For childhood patients with hypoglycaemia, hyperlipidemia, puberty delay and growth retardation, GSD should be considered. Gene sequencing is valuable for the quick identification of GSD subtypes.

## Background

Glycogen storage disease type I (GSD I), also known as von Gierk disease, is an autosomal recessive disorder resulting from the deficiency of glucose-6-phosphatase (G6Pase) activity [1]. G6Pase plays an important role in blood glucose (BG) regulation by catalyzing the final step of both glycogenolysis and gluconeogenesis together with the glucose-6-phosphate transporter, and its dysfunction will lead to the excessive accumulation of glycogen and fat in organs. The incidence of the disease is approximately 1 in 100,000 to 400,000 births accounting for about 25% of all types of glycogen storage disease [2]. The patients with GSD I mainly manifested as hepatomegaly, hypoglycemia, lactic acidemia, hyperlipidemia, hyperuricemia, puberty delay and growth retardation [3]. Abnormalities height, weight, BG, blood lipids can indicate the possibility of suffering from this disease.

Some clinical manifestations are similar in forms of GSD associated with liver, including GSD 0, GSD III, GSD IV, GSD VI, GSD IX, GSD XI, but the disease-causing genetic mutations and the treatment of them may be different. To improve the understanding of this disease, we report here a case of GSD Ia hospitalized in Southwest hospital in November, 2016. This study was approved by the ethics committee of Southwest Hospital, Chongqing, China. Informed consents were obtained from his parents. The study protocol conforms to the ethical guidelines of the 1975 Declaration of Helsinki.

## Case presentation

A 16-year-old male patient was admitted to the Department of Infectious Disease, Southwest Hospital for defining the nature of his space-occupying lesions in liver on November, 2016. He was diagnosed as hepatitis B around 1-year-old (his mother had hepatitis B, and did not do any mother to child blocking during pregnancy. So we deduced that his hepatitis B came from vertical transmission). The patient had not received any treatments due to the poor local medical condition, but he had regular visits in several hospitals. On June, 2015, he was found to have hepatomegaly and multiple space-occupying lesions in liver by ultrasonography, and was considered the possibility to suffer hepatocellular carcinoma.

Physical examinations showed this 16-year-old boy was 135 cm in height and 29 kg in weight, below the average values of peers. And his secondary sex characteristic was undeveloped. His liver could be palpable 2 cm below the right rib and 4 cm below the xiphoid, with rigidity and blunt edge. The marked percussion tenderness over liver region was present.

Imaging studies showed some evidence supporting hepatocellular carcinoma, as well as some evidence didn’t support it. Upper abdominal enhancement CT scan showed chronic liver disease performance and nodular low-density shadows in the left and right posterior lobe of the liver. Contrast-enhanced ultrasonography of the abdomen showed the space-occupying lesions with high central density and low density rings around. Significant enhancement of high central density was seen in arterial phase, persisting in portal phase and equilibrium phase. While low density rings enhancement was insignificant.

Laboratory examinations showed decreased blood testosterone (T, 0.12 ng/ml, reference range: 1.75–7.81 ng/ml), blood estradiol (E2, 2.00 pg/ml, reference range: 20–75 pg/ml), insulin-like growth factor-1(97.39 ng/ml, reference range: 224–592 ng/ml) and increased 8:00 cortisol (616.23 ng/ml, reference range: 66–286 ng/ml).

Tracing the clinical history of the patient, we found that he was admitted to hospital (the detail is unreachable) for growth retardation on July, 2015. The Bone age test indicated that the left hand development maturity score was 714, which equals to a 11.6-year-old male’s bone age. And the lab examinations were showed in Table 1. Then he began to receive intramuscular injection of growth hormone and oral lamivudine treatment. During the treatment, the liver function continued to be abnormal, and the growth hormone injection treatment was not effective.

**Table 1:** Primer sequence for G6PC gene amplification and sequencing

Exons	Forward primer	Start	Reverse primer	Start
5–1	cacctccatctgaaagagtc	c.643–200	gagtccacaggaggtctaca	c.792
5–2	acccacctctagcaaaggtc	c.643–64	gcaaagggtgtggtgtcaat	c.856

According to these evidences, he was suspected of suffering from glycogen storage disease type I instead of hepatocellular carcinoma.

To confirming the doubt of GSD I, the patient underwent the Gd-EOB-DTPA tumor specific examination and liver biopsy.

**Fig. 1:**
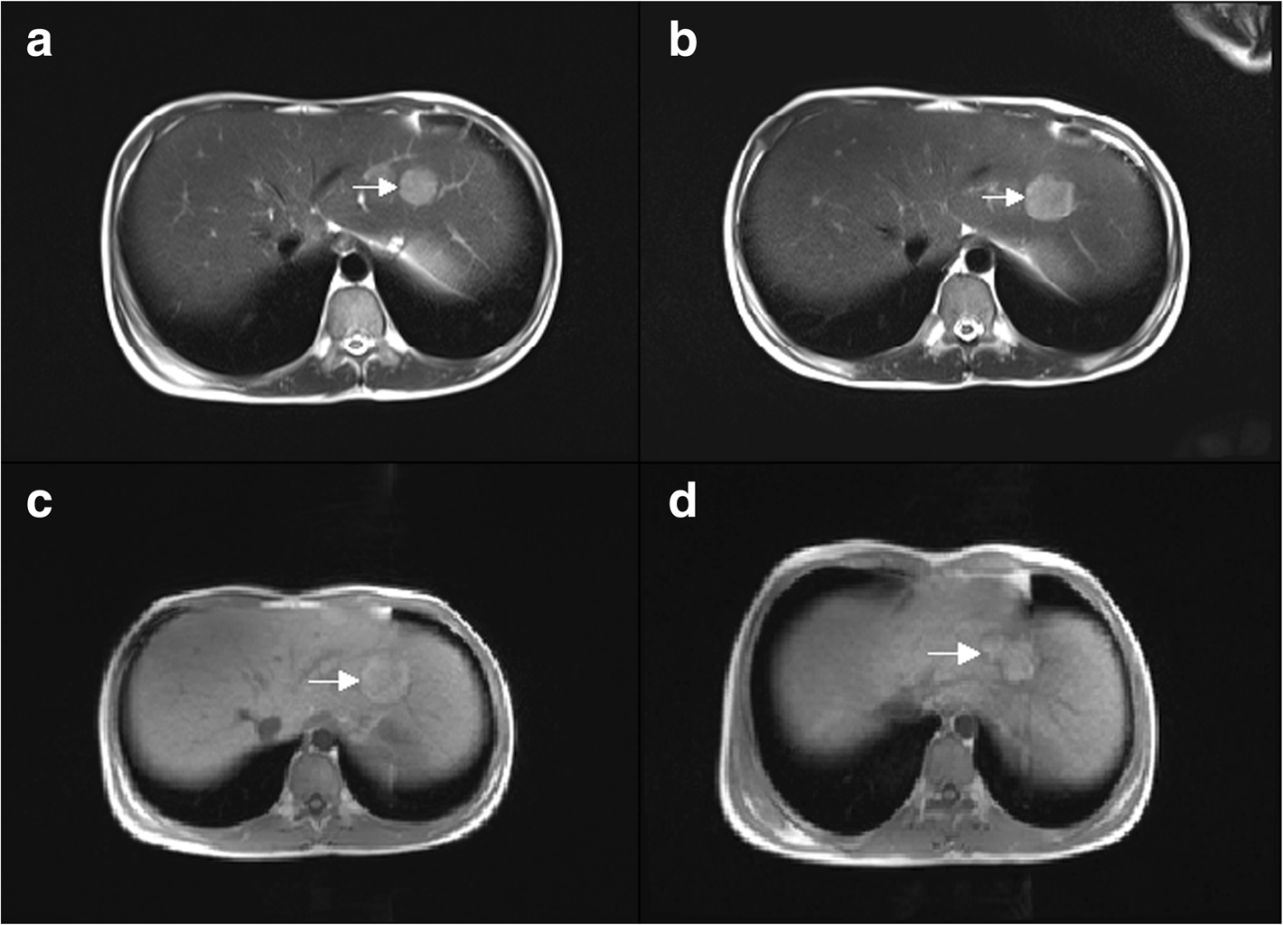
The Gd-EOB-DTPA tumor specific detection showed: glycogen accumulation. Arrow shows the largest nodule. **a** MRI (Gd-EOB-DTPA) examined on base line (2016.11.11 at presentation), the max size of nodules was 23 mm. **b** MRI (Gd-EOB-DTPA) examined 9 months after diagnosis of GSD I, the max size of nodules was 27 mm (**c**). MRI (Gd-EOB-DTPA) examined 12 months after diagnosis of GSD I, the max size of nodules was 34 mm. **d** MRI (Gd-EOB-DTPA) examined after radiofrequency ablation

**Fig. 2:**
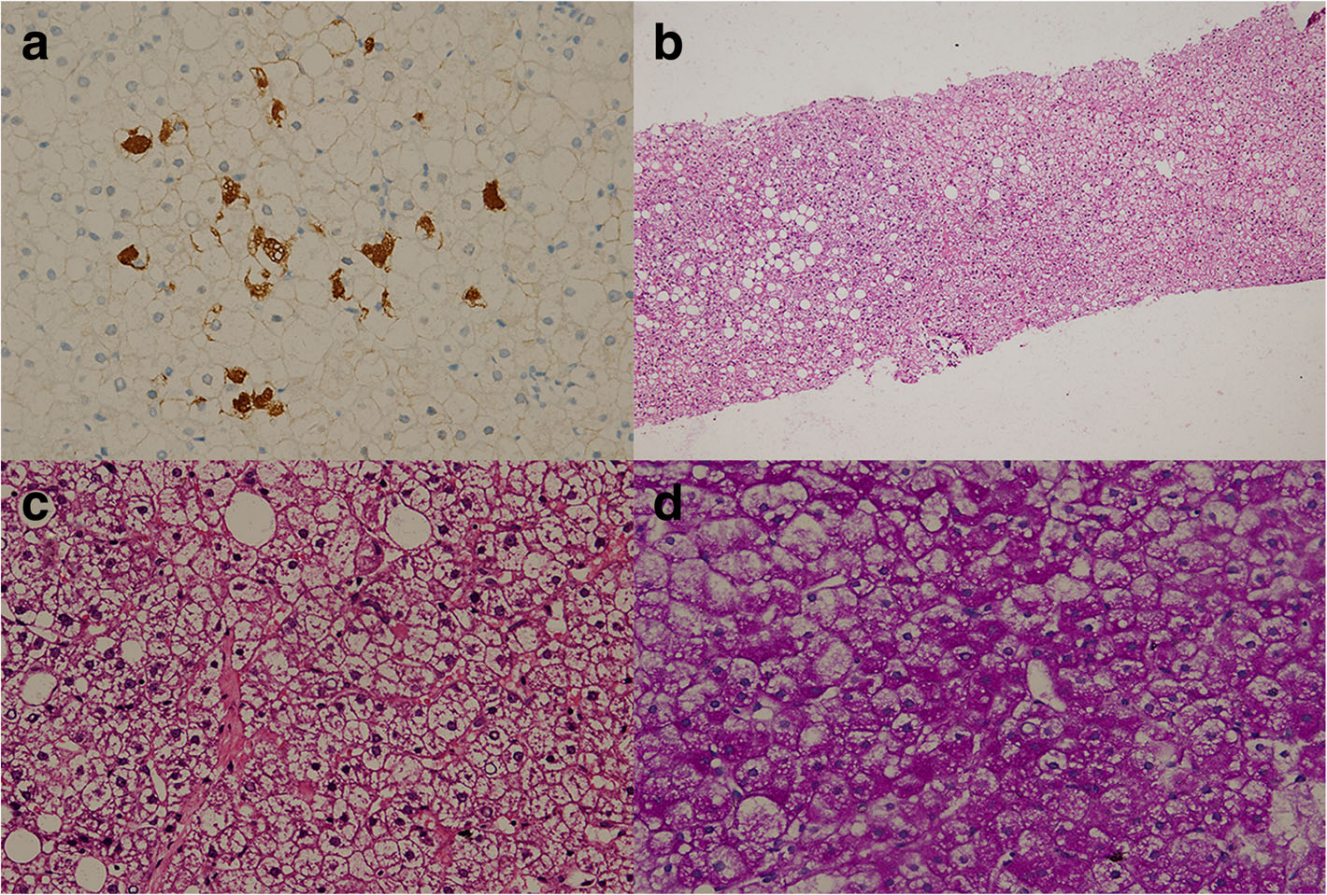
Pathological examination. **a** HBsAg staining of several cells was positive (400×). **b** HE staining of liver biopsy tissue (100×). **c** HE staining of liver biopsy tissue (400×). **d** PAS staining suggested a large deposition of glycogen in hepatocytes

The Gd-EOB-DTPA tumor specific examination suggested glycogen accumulation (Fig. 1). As shown in Fig. 2, the pathological examination results of the patient showed that the liver cells were marked swollen with fatty changes, and a small number of neutrophils infiltrated with fibroblasts. HBsAg staining of several cells was positive (Fig. 2a), HBcAg staining was negative, and PAS staining suggested a large deposition of glycogen in hepatocytes (Fig. 2d). The diagnosis was mild chronic hepatitis (G2S1) combined with glycogen accumulation.

There are 12 subtypes of glycogen storage disease, and their genetic variation, treatment, prognosis, diet intervention are different. So it’s necessary to confirm the subtype of glycogen storage disease.

After informing the patient and family, and obtaining the signed informed consent, we collected the peripheral blood of the patient and his father, mother and two sisters (one elder sister cannot be collected for marrying to other province), and extracted DNA from white blood cells. Due to various types of glycogen storage disease involving many genes, and nonspecific symptoms easily confused with other liver metabolic disease, so we first sequenced the entire exome of the patient to find the mutant gene, and then used first generation of sequencing to verify the mutation in the patient and his families. The exome sequencing applied Illumina Hi-seq using Agilent Surelect Kit, and the platform for Sanger sequencing is Applied Biosystems® 3730 DNA Analyzer by using BigDyeTM Terminator v3.1 Cycle Sequencing Kit.

**Fig. 3:**
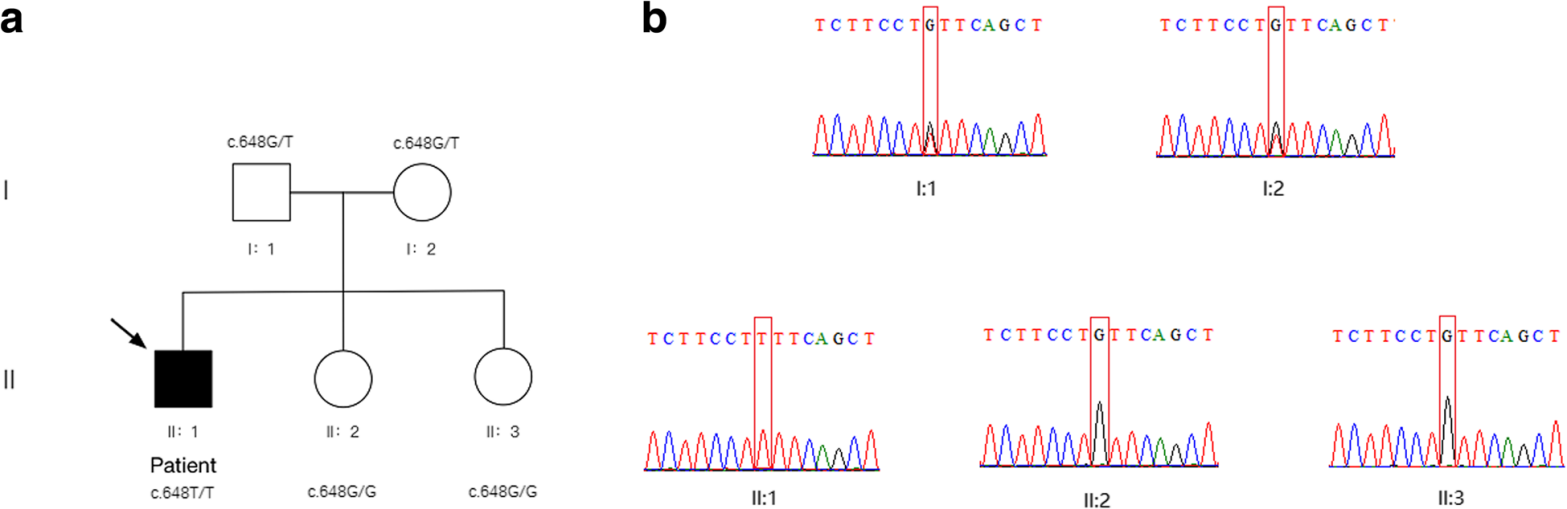
Mutational analysis in the patient pedigree. **a** The genotypes of G6PC gene for family members. Roman numerals indicate generations and Arabic numbers indicate individuals. Squares = males, circles = females. Affected individuals are denoted by solid symbols and unaffected individuals are denoted by open symbols. The index patient is indicated by an arrow. The two mutations were inherited from father and mother respectively. **b** Validation for the c.G648T of exon 5 by Sanger Sequencing. The red frame was mutation point

The result of exome sequencing suggests that there was a homozygous mutation c.G648 T (p.L216 L, NM_000151) on exon 5 of G6PC gene (rs80356484), which causes CTG changing to CTT at protein 216 and creates a new splicing site 91 bp downstream of the authentic splice site, though both codons encode leucine [4]. In order to confirm the sites and homozygosity of the mutations, we designed sequencing primers near mutation sites (the sequences of primers are shown in Table 1) and performed PCR for genome amplification of the patient and his families. The result of the first generation of sequencing of the patient is in accordance with exome sequencing, and the mutation c.G648 T was heterozygous identified in his father and mother. (Fig. 3). The mutation found on the G6PC gene is a mutation site of the glycogen storage disease type Ia, which has a high frequency in the population of Chinese and Japanese patients with glycogen storage disease type Ia [4, 5].

According to clinical manifestations, auxiliary examinations, tissue pathology and genetic testing, this patient was diagnosed as a GSD type Ia complicated with hepatic adenoma, and combined with chronic hepatitis B.

The patient was treated by corn starch treatment (Corn starch 50~100 g, 4 times a day) and practiced low fat diet immediately after GSD was suspected. Since then, he was followed up regularly in Department of Infectious Department of Southwest Hospital. The examination data of the patient were shown in Table 2.

**Table 2:** Examination data on presentation and follow-up

Variable	Reference range	Baseline	9 Months	12 Months	18 Months
Blood glucose (mmol/L)	3.9–6.4	3.19	3.58	4.15	3.43
Triglyceride (mmol/L)	0.4–1.73	5.76	4.17	3.32	2.98
Total cholesterol (mmol/L)	3.1–5.7	5.51	5.54	6.57	5.95
LDL-cholesterol (mmol/L)	2.07–3.1	4.06	3.94	4.49	4.06
HDL- cholesterol (mmol/L)	0.9–2.0	1.45	1.6	2.07	1.89
Testosterone (ng/ml)	1.75–7.81	0.78	2.55	2.78	1.8
Uric acid (μmol/L)	155–428	493.2	639.2	509.5	580.2
Creatine (μmol/l)	25–104	33.2	36.5	41.6	33.9
Urea (mmol/L)	1.7–8.3	2.5	1.7	4.6	3.4
Growth hormone (ng/ml)	0.55–4.47	0.62	–	3.73	25.24
Urine Ketone	negative	Weakly positive	–	Weakly positive	–
Height (cm)	–	138	152	155	–
Weight (kg)	–	29	35.8	36	–
BMI	–	15.23	15.50	14.98	–
Hepatic adenoma(n, min, max(mm))	–	7, 7, 23	7, 10, 27	7, 11, 34	7, 12, 34
Corn starch therapy	–	Started taking	not follow the scheduled treatment for last 3 months	Continuously taking	Continuously taking
Radiofrequency ablation	–	–	–	Done	–

During follow-up, the patient stopped corn starch diet for about three months, and the laboratory measures showed deterioration in July, 2017.

Unexpectedly, we found that the size and the number of hepatic adenomas were increasing during the follow-up. In November, 2017, the patient had an indication for surgery as MRI imaging showed the largest one of hepatic adenomas had reached 3.1 × 3.4 cm, he was therefore admitted to the Department of Hepatobiliary Surgery, Southwest Hospital for radiofrequency ablation and liver biopsy. The postoperative pathological result confirmed as hepatic adenoma.

## Discussion and conclusions

This case was diagnosed as GSD type Ia complicated with hepatic adenoma and combined with chronic hepatitis B.

The patient showed the most significant characteristic of GSD type I, growth retardation, because kidney injury causes the uric acid depositing in the joints, resulting in osteoporosis [6]. Although growth hormone deficiency can also lead to growth retardation, this GSD I patient appeared hypoglycemia and hyperlipidemia at the same time, which is a strong signal that suggests GSD I.

Some of GSD I patients will manifest diarrhea and skin cholesterol deposition (xanthoma) with age [7]. Other clinical characteristics include doll-like facies, poor growth, short stature, and a distended abdomen due to pronounced hepatomegaly and nephromegaly [1]. There is also possibly ovarian dysplasia(PCOS)happen in female patients [8] and adenoma forming in liver appear in teenager patients, the adenoma can occasionally cancerous [9].

The genetic variation of the patient reported here is a homozygous point mutation at the exon 5 of G6PC gene at 17th chromosome, c.G648 T (p.L216 L, NM_000151, rs80356484). This pathogenic mutation causes CTG changing to CTT at protein 216. Though both codons encode leucine, this silent mutation creates a new splicing site 91 bp downstream of the authentic splice site, and has a high frequency in the population of Chinese and Japanese glycogen storage disease type Ia patients [5].

This case was diagnosed as GSD type Ia complicated with hepatic adenoma, combined with hepatitis B based on: (1) HBsAg positive and HBV-DNA positive; (2) clinical manifestations: growth retardation, facies hepatica, hepatolienomegaly; (3) laboratory examination: fasting hypoglycemia, postprandial hyperglycemia, transaminase abnormality, hyperlipidemia, hyperuricemia, and no neutrophils deficiency; (4) imaging examination: Gd-EOB-DTPA MRI indicated glycogen accumulation in the liver, and ultrasonography considered fatty liver and hepatic adenoma; (5) liver biopsy: PAS staining suggested a large deposition of glycogen in hepatocytes; (6) gene detection: G6PC gene mutation, and related reports existed; (7) no evidence of other types of liver disease.

However, the deficiency of our report is that there was no activity detection of related enzymes, especially the enzyme activity detection of liver tissue. After receiving corn starch therapy, the liver adenomas are still increasing, and we did not find any cause to explain this phenomenon.

This patient was diagnosed as GSD Ia combined with chronic hepatitis B, who responded to corn starch intervention. For childhood patients with hypoglycaemia, hyperlipidemia and growth retardation, GSD should be considered. Before the cause of growth retardation is identified, the application of growth hormone should be careful. Although gene sequencing is valuable for the quick identification of GSD subtypes, it is usually not required when suspicious clinical and biochemical evidences are apparent. If we encounter adolescent chronic hepatitis B patients with growth retardation, hypoglycaemia and hyperlipidemia, we must consider diagnosis of GSD as soon as possible, to avoid misdiagnosis due to inertial thinking. When GSD I is suspected, treatments should be started without any delay before confirmation tests are applied.

## Abbreviations

CHB: Chronic hepatitis B
GSD: Glycogen storage disease
SNP: Single nucleotide polymorphism
